# Symptomatic Female Genital Tract Infections Due to *Neisseria meningitidis* in Athens, Greece

**DOI:** 10.3390/diagnostics11071265

**Published:** 2021-07-14

**Authors:** Anastasios Tsakalos, Athanasia Xirogianni, Georgia Ekonomou, Anastasia Papandreou, Efstratios Prokopiou, Eleni Vagiakou, Georgina Tzanakaki

**Affiliations:** 1 Microbiology Department, “Georgios Gennimatas” General Hospital of Athens, 11527 Athens, Greece; elenivagiakou@gmail.com; 2 National Meningitis Reference Laboratory, Department of Public Health Policy, School of Public Health, University of West Attica, 11521 Athens, Greece; axirogianni@uniwa.gr (A.X.); npapandreou@uniwa.gr (A.P.); gtzanakaki@uniwa.gr (G.T.); 3 Gynecology Department, “Georgios Gennimatas” General Hospital of Athens, 11527 Athens, Greece; ikonomoug@hotmail.com (G.E.); n.orlgyn@gna-gennimatas.gr (E.P.)

**Keywords:** *N. meningitidis*, unusual presentations, female genital tract infections, serogroups, molecular typing, MLST

## Abstract

*Neisseria meningitidis* is considered as an obligate human pathogen and can cause life-threatening diseases like meningitis and/or septicaemia. Occasionally, it can be recovered from infections outside the bloodstream or central nervous system, like respiratory, ocular, joint, urogenital or other unusual sites. Herein, we present two rare cases of female genital infections due to *N. meningitidis* within a two-year period (2019–2020), identified as serogroup B (MenB) and Y (MenY), respectively. Genotypic analysis for PorA, FetA and MLST revealed the following characteristics: MenB: 7-12, 14, F5-36, 1572cc and MenY: 5-1,10-1, F4-5, 23cc, respectively. Such unusual presentations should alert the clinicians and microbiologists not to exclude *N*. *meningitidis* from routine diagnosis and the need of early detection. This is the first report in Greece, and, to our knowledge, in Europe since 2005 describing meningococcal female genital infections.

## 1. Introduction

*Neisseria meningitidis* or meningococcus is a beta proteobacterium of the *Neisseriaceae* family and an obligate human pathogen [1]. It is considered to be a transient member of the oro- and nasopharyngeal flora and person-to-person transmission occurs via respiratory secretions. According to their highly virulent polysaccharide capsule, thirteen serogroups have been recognised, while only six (A, B, C, W-135, Y, and X) are responsible for the majority of cases of invasive meningococcal disease (IMD) worldwide [2].

*N. meningitidis* is estimated to be carried by almost 10% of the population, while in industrialised countries carriage rate can be as high as 23.7% in adolescents and young adults [3]. Even though it is usually asymptomatic, the mucosa colonisation can lead to bloodstream invasion and subsequently life-threatening IMD in otherwise healthy individuals, like meningitis and/or septicaemia [4].

Rarely, meningococcus can be recovered from unusual sites causing various infections outside the bloodstream or central nervous system, such as respiratory [5], ocular [6], joint [7], and urogenital [8].

*N. gonorrhoeae* or gonococcus, causing gonorrhoea, a sexually transmitted disease, is also considered an obligate human pathogen. Although *N. meningitidis* and *N. gonorrhoeae* are thought to occupy distinct human ecological niches, a literature search reveals various reports are indicating that *N. meningitidis* can potentially invade the human genital tract, even though historically it is not considered a common genital pathogen. However, its isolation from genital samples was recognised almost 80 years ago, by the first reported cases of male meningococcal urethritis in 1939 [9], while first reports associating *N. meningitidis* with female genital tract infections appeared a few years later [10,11]. According to the literature, 129 cases of meningococcal female genital infections were reported worldwide out of which 89 cases were found in the USA. In contrast, 24 cases have been reported in Europe so far, the majority being diagnosed during the 1990s (14 cases), while none have been reported since 2005 [12,13], a fact that possibly reflects the low frequency of these infections. 

The transmission route is controversial and usually by orogenital contact with a sexual partner carrying the microorganism in the upper respiratory system which may play a crucial role [8]. The majority of the cases are seen in adult women [14] or adolescents [15], suggesting cross-colonisation of sexual partners. Phenotypic and genotypic methods approached by some studies with the use of pulsed field gel electrophoresis (PFGE) revealed identical isolates in both the oropharynx and genital systems [13,16]. Herein we present two rare cases of symptomatic female lower genital tract (LGT) infections within a two-year period (2019 and 2020) in Greece, in which *N. meningitidis* was isolated during a routine microbiological investigation that was first suspected to be *N. gonorrhoeae*.

## 2. Materials and Methods

In both cases, otherwise healthy heterosexual females of 22 (case 1) and 53 (case 2) years of age, respectively, attended the gynaecology outpatient departments of two different tertiary hospitals in Athens, Greece, during the period 2019–2020, describing increased vaginal discharge. Routine gynaecological examination was performed and optimal genital samples and smears were obtained for routine microbiological investigation including microscopy (wet and Gram-stained smears), culture, a genital mycoplasmas’ detection test and a direct fluorescent antibody test for *Chlamydia trachomatis.*

The specimens were inoculated in blood, chocolate, chocolate PolyViteX VCA3 (bioMérieux, Marcy l’Etoile, France) and Sabouraud Gentamicin Chloramphenicol 2 (SGC2) (bioMérieux, Marcy l’Etoile, France) agar plates, according to the standard microbiological protocols for genital samples. Chocolate and VCA3 agar plates were incubated at 37 °C in the presence of 5% CO_2_ conditions while blood and SGC2 agar plates were under aerobic conditions at 37 °C.

Initial colony identification was performed by conventional methods (Gram stain, oxidase test) while further identification was carried out by a VITEK 2 automated system (bioMérieux, Marcy l’Etoile, France). In addition, the supportive use of BioFire FilmArray multiplex PCR system with Meningitis/Encephalitis Panel (BioFire Diagnostics, Salt Lake City, UT, USA) was employed, even though this panel is exclusively evaluated mainly for cerebrospinal fluid samples.

Phenotypic and genotypic identification was performed at the National Meningitis Reference Laboratory, School of Public Health, University of West Attica. Serogroups were identified by a slide agglutination test (Remel Europe Ltd., Dartford, Kent, UK.) according to the manufacturers’ instructions and by multiplex PCR targeting specific capsule group genes (A, B, C, W, and Y) as described previously [17].

Furthermore, the isolates were characterised by ‘finetyping’ (MLST, PorA, and FetA), as described previously [18,19] using the PubMLST.org *Neisseria* database (http://pubmlst.org/neisseria/, accessed on 18 March 2020 and 05 March 2021 respectively) [20]. Sequence types (ST) were defined and grouped into clonal complexes (ccs). PorA genotyping for variable regions 1 and 2 (VR1 and VR2) was performed as described previously [18] and compared with the sequences in the PorA *Neisseria* database (http://pubmlst.org/neisseria/PorA/, accessed on 18 March 2020 and 05 March 2021 respectively) [20]. Similarly, the FetA variable region (FetA VR) was also determined, as previously described [21], and compared with FetA VR sequences in the *Neisseria* database (http://pubmlst.org/neisseria/FetA/, accessed on 18 March 2020 and 05 March 2021 respectively) [20].

Antimicrobial susceptibility testing determining the Minimum Inhibitory Concentration (MIC) for seven antimicrobial agents (penicillin, cefotaxime, ceftriaxone, rifampicin, chloramphenicol, cefaclor and ciprofloxacin) was performed by the E-test strips (LIOFILCHEM S.R.L, Roseto degli Abruzzi, Italy), according to EUCAST recommendations.

## 3. Results

Clinical examination revealed mild LGT infection. Microscopically, numerous polymorphonuclear leucocytes were detected along with intra- and extracellular Gram-negative coffee-bean shaped diplococci. On 24 h incubation, small, round, greyish, smooth colonies were observed on both chocolate and VCA3 agar. The colonies were oxidase-positive, Gram-negative coffee-bean shaped diplococci. In both cases, the isolates were finally identified as *N. meningitidis* by VITEK 2. Furthermore, the meningococcal genetic material was also detected by the BioFire FilmArray system.

After further identification by serogroup, both isolates were found to belong to serogroups B (MenB) and Y (MenY), respectively. Genotypic analysis for PorA, FetA and MLST clonal complex revealed the following characteristics: MenB: 7-12, 14, F5-36, 1572 cc and MenY: 5-1, 10-1, F4-5, 23 cc, respectively (Table 1).

Both isolates expressed reduced susceptibility to penicillin, resistance to chloramphenicol while they were susceptible to the rest of the antimicrobial agents tested.

There was no clinical evidence of a meningococcal systemic infection in any of the two cases. Both cases were treated with cefaclor orally and the follow up culture in case 1 was negative. Pharyngeal samples were not obtained neither from the cases nor from the sexual partners and appropriate chemoprophylaxis was not prescribed to close contacts. In contrast, follow up culture was not performed in case 2.

## 4. Discussion

In our study, the symptoms in both cases were presented by increased vaginal discharge which is in agreement with previous studies at which meningococcal invasion of the female genital tract can cause local symptoms, possibly due to cervicitis or vaginitis [22], or can be asymptomatic, identified only during routine follow-up examination [23]. In contrast, vaginal bleeding [24], abdominal symptoms due to pelvic inflammatory disease [25] or systemic symptoms due to IMD [26] were not observed. Previous study describes a case in which the patient developed fulminant meningitis secondary to symptomatic meningococcal vaginitis [27]. 

During the 80-year period (1940–2020) of recorded meningococcal female genital infections, all cases were sporadic and the frequency of these infections was low, especially in Europe. Since 2005 no cases have been reported in Europe ground as well as no epidemics or outbreaks being recorded so far. On the contrary, *N. meningitidis* appears to be a more frequent cause of male urethritis [8]; while, since 2015, an outbreak of meningococcal urethritis primarily among heterosexual men was reported in many US cities [28], with a study in Columbus, Ohio describing 75 cases between January and November of 2015 [29]. These cases were attributed to the emergence of a novel clade of NonGroupable (NG) *N. meningitidis* within the ST-11 clonal complex, the “U.S. NmNG urethritis clade”, which has been recently reported also in cases in the UK [30]. This well studied clade is believed to possibly be adapted to the male urogenital environment with the insertion of IS1301 with the associated deletion of the capsule, enhancing mucosal adherence, and the acquisition of the gonococcal denitrification pathway by gene conversion, promoting anaerobic growth [31]. 

In contrast, this specific clade is not detected among those of the female genital tract infections for the cases which serogroup identification was available. Specifically, among 36 cases serogroups B (MenB) and C (MenC) were the most prevalent, accounting for 45% (16/36) for Men B and 27% (16/36) for Men C, respectively, while only four isolates belonged to serogoup Y. However, gentotypic information was not provided in any of the studies, in contrast to our study in which the strains were fully ‘finetyped’.

Both meningococcal isolates expressed reduced susceptibility to penicillin suggesting a possible transmission of penicillin-resistance genes from *N. gonorrhoeae* to *N. meningitidis*, since resistance to penicillin is common in *N. gonorrhoeae* as gene flow between the two *Neisseria* species were detected [32]. This is of great concern, as previous studies suggest [33].

As *N. gonorrhoeae,* and recently although rare, *N. meningitidis,* can colonise in the female genital tract, being able to cause similar clinical manifestations and implications and expressing similar characteristics in microscopy, culture growth and conventional identification tests [34], a possible misidentification should be considered. For this reason, clinical and microbiological suspicion along with accurate identification methods including molecular tests is necessary for appropriate diagnosis, treatment and public health purposes, like prophylaxis of direct contacts. Additionally, isolates’ genotypic characterisation is of great importance for detailed molecular epidemiological investigation and surveillance of *N. meningitidis* in similar cases.

To our knowledge, this is the first report in Europe since 2005 describing symptomatic female genital tract infections due to *N. meningitidis*. The two rare cases, being also the first described in Greece within a two-year period (2019–2020), should alert clinicians and microbiologists for a possible increase in similar cases and the need for early detection. Furthermore, this appears to be the first study of meningococcal female genital infections in which the isolates were fully genotypically characterised. 

## 5. Conclusions

In conclusion, *N. meningitidis*, although rare, can be recovered from unusual sites and specimens. Clinicians and microbiologists should always be aware and consider meningococcus as a potential causative agent of infections other than meningitis and septicaemia. 

## Figures and Tables

**Table 1:** Meningococcal characteristics of the two cases.

	Case 1	Case 2
Serogroup	B	Y
PorA	7-12, 14	5-1, 10-1
FetA	F5-36	F4-5
MLST	ST 15543, 1572cc	ST 1655, 23cc
MIC (mg/L)		
Penicillin	0.125	0.5
Chloramphenicol	3.0	3.0
Cefaclor	0.019	0.75
Cefotaxime	0.012	0.006
Ceftriaxone	0.004	0.004
Rifampicin	0.012	0.047
Ciprofloxacin	0.004	0.004

## Data Availability

Not applicable.
